# Work smart, not hard: analysis of delays faced by clinical trials investigating spinal fusion using Protocol AI

**DOI:** 10.3389/fsurg.2025.1546367

**Published:** 2025-03-27

**Authors:** Katia Schiegg, Philipp Khlebnikov, Florian Meer, Joel Kühl, Poorya Amini, Janine Antonov, Emin Aghayev, Stephan Radzanowski, Quentin Haas

**Affiliations:** ^1^ Research Centre, Lindenhofgruppe AG, Bern, Switzerland; ^2^ Risklick AG, Spin-off University of Bern, Bern, Switzerland; ^3^ Orthopädie Sonnenhof, Bern, Switzerland

**Keywords:** artificial intelligence, spinal fusion, clinical trial, Risklick, Protocol AI

## Abstract

**Introduction:**

Degenerative diseases of the spine are increasingly prevalent with age. Spinal fusion is a common treatment if non-invasive or less-invasive treatment approaches have not been successful. Numerous clinical trials on spinal fusion are started every year to investigate novel technologies worldwide. However, a substiantial amount of trials are terminated prior to completion.

**Research question:**

In this study, we analyzed the historical performance of all clinical trials on spinal fusion since 2010.

**Material and methods:**

The identification of related trials was carried out using Protocol AI, which is the Risklick's software. It collects and updates clinical trial data from various sources, including clinical trial registries and datasets from the World Health Organization. Protocol AI has automatically extracted the data on trial, categorized them, and clustered them in trial phases.

**Results:**

The historical probability of early termination for a clinical trial investigating spinal fusion was approximately 25%. The average trial delay for completed trials was 10.6 months. With an average anticipated trial duration approaching 40 months, the observed delay represents an extension of 25% of the anticipated trial duration for completed trials. Trials facing delay and failure predominantly reported critical issues with patient recruitment.

**Dicsussion and conclusion:**

This study emphasizes the importance of implementing a strict risk management plan and recruitment plans, while suggesting professionals to implement standardized enrollment monitoring analyzes during the course of the trial. The amelioration of recruitment policies could substantially maximize the performance of trials within the field, benefiting patients and all stakeholders involved.

## 1 Introduction

The spine is a complex and central load-bearing structural element of the musculo-skeletal system, enabling the motor and sensory functions of the human body (1). The spine is affected by time and the signs of wear become apparent through degeneration. Degenerative diseases of the spine are very common and are increasingly prevalent with age (2). A key procedure to treat degenerative diseases of the spine (among other interventions) is a spinal fusion (3). However, despite the technological advancements in the field in the last two decades, there are suggestions that up to a third of patients are not satisfied with the results after spinal fusion surgery (4, 5).

The combination of these facts, along with the previously mentioned high prevalence and the continuous ageing of society, underscores the significance of research on spinal fusion surgery in today's world of orthopedics. However, research including clinical trials is a resource intensive undertaking. Moreover, the combined costs pose a significant burden for researchers and can contribute to the unsuccessful conclusion of a clinical trial (6). The complexity of clinical trials has surged over the years, emerging as a significant challenge to their success (7). This heightened complexity is closely correlated with rising average costs and an escalated risk of failure (8). Despite the pivotal role that trial delays play in costs and success probability, this aspect remains inadequately studied (6).

The aim of the study was to investigate the planned performances of the trials and contrast them with the actual trial outcomes to identify the probability for their termination as well as reasons for delays.

## 2 Materials and methods

To investigate these issues, we utilized Protocol AI, a state-of-the-art trial analytics software developed by Risklick, to compile all clinical trials conducted in the field of spinal fusion and analyze the data using its fully automated technology.

### 2.1 Data collection

Protocol AI collects and updates clinical trial data weekly from various sources, including clinical trial registries and datasets from the World Health Organization (WHO). Protocol AI's power and accuracy in retrieving relevant trials have been validated in peer-reviewed publications (9, 10). Protocol AI additionally collects and updates metadata from various sources such as PubMed, Embase, BioRxiv, and MedRxiv, including publication titles, abstracts, journal names, publication dates, and digital object identifier (DOI) numbers.

### 2.2 Technology

Protocol AI preprocesses all clinical trial and publication data to conform to a predefined data format. It then adds the data to Elasticsearch, performing analysis as a full-text search and analytics engine for both clinical trials and publications. Protocol AI normalizes indexed data and queries using a text preprocessing pipeline that includes technologies such as tokenization, lowercasing, stop-word removal, and stemming. The Elasticsearch cluster is responsible for maintaining the indices.

The index model parameters are tuned using a set of manually annotated queries. The similarity measure is computed using the divergence from randomness model (DFR) with the term frequency normalization set to 20.0 (11). A detailed description of the pipeline is provided by Ferdowsi et al. (12).

We use a spinal fusion-specific ontology of standardized medical terms, synonyms, classes, and subtypes engineered by clinical trial domain experts to expand the user query and increase the recall of relevant documents (13).

### 2.3 Experimental setup

Regarding interventional clinical trials conducted on the topic of spinal fusion, a search was conducted for clinical trials that were started between 2010 and the search date (11th December 2023) with standard medical terms and synonyms for the query. The following key words were used: “spine fusion”, “fusion of the spine”, “spinal fusion”, “spine interbody fusion”, “vertebral fusion”, “vertebra fusion”, “vertebrae fusion”, “fusion of the vertebrae”, “fusion of the vertebrae”, “interbody fusion”, “spondylodesis”, “anterior fusion”, “posterior fusion”, “posterolateral fusion”, “postero-lateral fusion”. The search identified 595 clinical trials, With duplicates removed using the Risklick tool named Deduklick (14).

### 2.4 Data validation and selection criteria

Following the search, unsuitable clinical trials were eliminated through the following steps (see flowchart in Supplementary Figure S1). First, a score (0–100) was automatically assigned by Protocol AI to each trial based on relevance and proximity of the clinical trial and the search criteria. The accuracy and specificity of the scoring system were validated in parallel to the development of Protocol AI, confirming the solution's capabilities for the analysis of both clinical trials and clinical trials-related publications (9, 15). After data collection, all clinical trials with a score below 50 (representing >90% of all identified trials; *n* = 610) were manually reviewed by two individual experts for relevance and eligibility. All trials not directly involving spinal fusion or including patients undergoing other surgeries (like hip or knee surgery) were excluded (*n* = 33), confirming an accuracy of 94.6% for Protocol AI.

Furthermore, based on the assumption that protocols and trial processes vary from pediatric to adult clinical trials, clinical trials including pediatric patients (maximum patient age ≤ 25 years or minimum patient age < 15 years; *n* = 73) as well as trials with the focus on adolescent scoliosis (*n* = 2) were excluded.

Since the EU clinical trials register does not provide data on phase 1 clinical trials, the completeness of data could not be ensured. Therefore, the phase 1 trials were excluded from further analysis (*n* = 19). Additionally, pre-clinical trials, trials with unknown phase and those registered with multiple phases (phase 1–2, phase 2–3) were also excluded (*n* = 41) to enhance the robustness of the trial comparison.

The final sample comprised 427 clinical trials investigating adult patients undergoing spinal fusion that started between 2010 and the search date (11th December 2023). When analyzing variables associated with the selected trials, those presenting corrupted data were automatically removed by Protocol AI. An inclusion overview is available in Supplementary Figure S1, summarizing the trial selection and validation process.

### 2.5 Statistics and data analysis

Protocol AI automatically clustered the clinical trials into five phases (1, 2, 3, 4, not applicable). The FDA has defined the “not applicable” phase for clinical trials as studies performed on devices or with behavioral interventions (16). The clinical trials were then grouped based on their status: active, suspended, unknown, completed, terminated and withdrawn.

The following sub-categories were analyzed:

– sub-cohort 1—terminated trials, and
– sub-cohort 2—completed trials (see flowchart in Supplementary Figure S1)

The reported causes of trial termination in sub-cohort 1 were automatically classified by Protocol AI into the following nine categories: insufficient funding, safety parameters, PI left institution, regulatory decision, interim analysis, objective not met, COVID-19, low enrollment, and other causes.

For the analysis of sub-cohort 2 with completed trials, Protocol AI extracted data on planned sample size, representing the number of participants anticipated to participate in the study at the beginning of the trial. Additionally, it extracted data on final sample size, representing the number of participants reported at the end of the trial. Protocol AI also extracted the planned study duration, representing the anticipated duration of the trial at the beginning of the trials, and the final study duration, representing the final trial duration reported at the end of the completed trial (when available). Delay in trial duration for completed trials was calculated as the difference between final trial duration of completed trials and the planned study duration. Protocol AI also collected the planned primary duration, representing the anticipated duration of the trial at the beginning of the trials to collect the last data from the last enrolled participant, and the final primary duration, representing the actual time taken by each trial to obtain the last data point from the last enrolled participant. If the calculated delay was greater than three times the standard deviation, the trial was considered to be an outlier and excluded from graphs to improve their readability. The trial location was extracted as well.

The probability density function of the delay was computed using Gaussian kernels from Python package SciPy version 1.10.1. Visualizations were created using Python package Plotly version 5.6.0. No other statistical analysis was performed.

Regarding other variables associated with trials, primary outcomes from clinical trials of all sub-cohorts were automatically collected using Protocol AI. Protocol AI further clustered outcomes into groups using its criterion tokenizer and embedded them using Large Language Models (LLMs) (17). The consistency of the outcome classification by Protocol AI was manually reviewed and validated by two independent experts. The outcomes were embedded into numerical vectors for analysis, utilizing the SentenceTransformer model “all-MiniLM-L6-v2”. Protocol AI further analyzed these vectors, clustering them to reveal underlying patterns and similarities. The final step applied t-distributed Stochastic Neighbor Embedding (t-SNE) from the Python package Scikit-learn version 1.2.1 for dimensionality reduction. This step enabled the creation of a two-dimensional visual representation of the clustered outcomes, making the data more interpretable and accessible for further analysis. Experts then manually clustered the outcome groups into three overarching themes, referred to as cliques 1–3: (1) patient-reported outcome measures, (2) treatment and procedural parameters, and (3) physiological and functional health parameters.

Completed trials (sub-cohort 2) with phase “Not Applicable” were selected due to their predominance among other trial subgroups (*n* = 92) and subsequently analyzed for recruitment and delay assessments. Beyond the power and precision of the software, the entire analytical process—following expert validation of the trials—was performed within seconds, offering a significant advantage over conventional semi-manual methods typically used for systematic reviews.

## 3 Results

### 3.1 Spinal fusion trials: phases, status, and cause of termination

**Figure 1:**
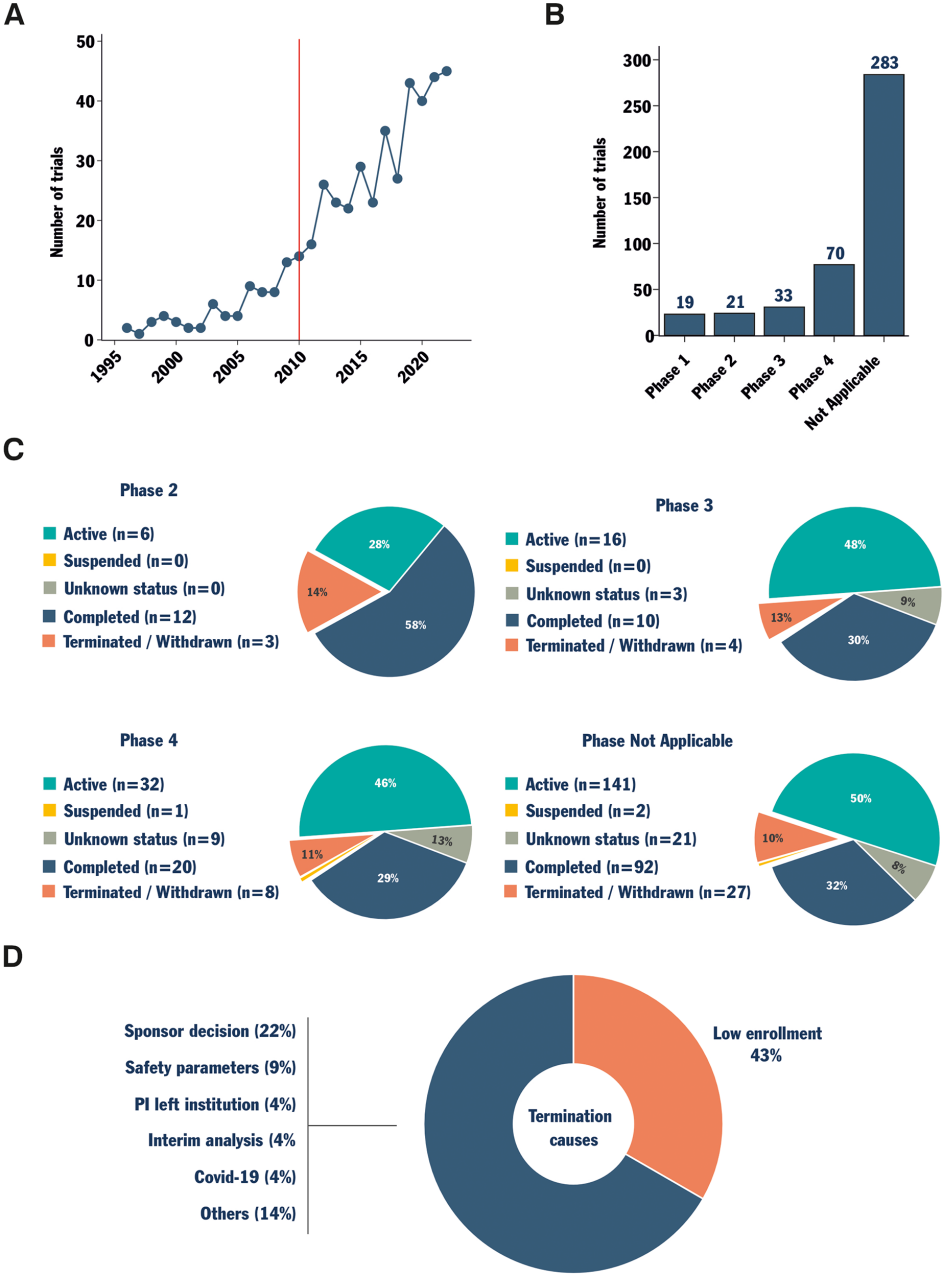
Data collection, status, and cause of termination analysis for trials investigating spinal fusion. Protocol AI collected spinal fusion-related clinical trials. The trials starting date was analyzed, and a red line marks the date from which trials were considered eligible for analysis **(**2010, **A)**. The trials were then sorted by phase **(B)**. Trials registered as phase 2, 3, 4, and not applicable phase were further analyzed based on their status **(C)**. Finally, the combined reported causes of termination for trials in phase 2, 3, 4 and the not applicable phase were analyzed **(D)**.

In the past decade, clinical research in the field of spinal fusion has shown consistent growth (Figure 1A). Trials were retrieved using Protocol AI. When examining trials initiated after 2010, a significant portion of trials were registered with no applicable phase (“Not Applicable”, Figure 1B). This category includes trials without FDA-defined phases, such as those involving devices or behavioral interventions, suggesting an increase in the development of novel devices for spinal fusion among all trials. Simultaneously, only a small fraction of the clinical trials identified were registered as phase 1, 2, or 3, with phase 4 trials representing the second most common group.

The status of each clinical trial was then investigated within each phase group, except for phase 1 trials due to an insufficient amount of data. Interestingly, apart from phase 2 trials, the proportions of trials with different statuses were similar for the trials in phases 3, 4 and “Not Applicable” (Figure 1C). These trial groups exhibited a proportion of terminated/withdrawn trials of between 10%–13% and a proportion of completed trials between 29%–32% (Figure 1C). When active trials and trials with unknown status were excluded, the proportion of terminated (failure-to-completion) trials approached one in four. In other words, excluding the few phase 1 and phase 2 trials, the historical probability of early termination for a clinical trial investigating spinal fusion was approximately 25%. Analyzing the officially reported causes of termination for terminated and withdrawn trials revealed that 43% of them reported issues with enrollment (Figure 1D). This reported cause of termination surpassed all others, underscoring persistent challenges in patient recruitment in the field.

### 3.2 Participant recruitment and trials delay

**Figure 2:**
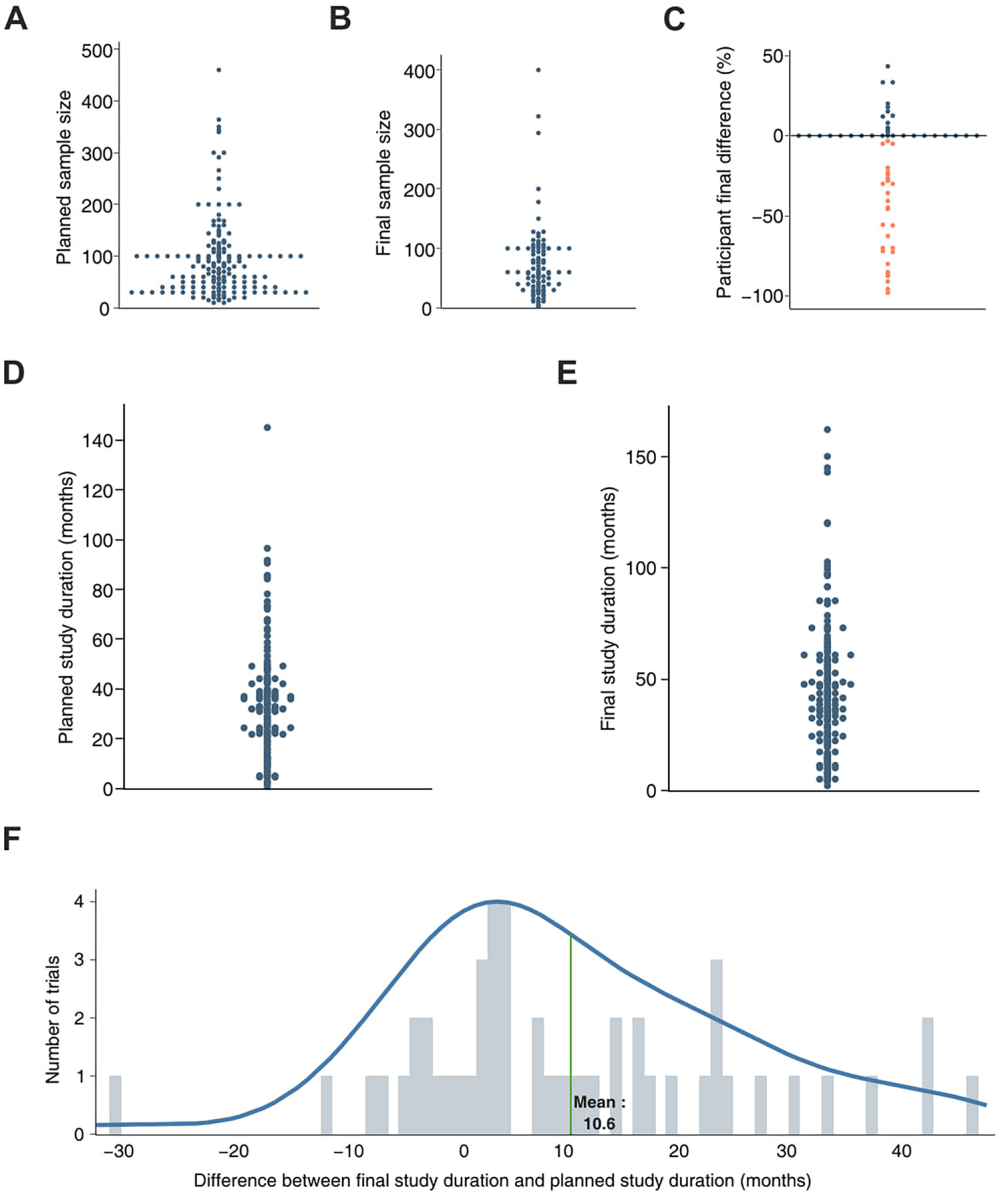
Analysis of sample size, trial duration and delay to reach trial completion date of trials registered with not applicable phase. Protocol AI further analyzed the trial variables previously collected and plotted the planned sample size (sample size selected prior to trial start, **A)** and the final sample size (sample size reported at the end of the trial, **B)** of trials with not applicable phase. The difference between the planned and final sample sizes was calculated for each trial and presented as a percentage of the estimated sample size **(C)**. The same approach was applied to the planned study duration (study duration selected prior to study start, **D)** and the final duration of the trial (duration reported at the end of the trial, **E)** The difference between the planned duration and the final duration of the trial led to the estimation of the delay faced by the trials, expressed in months **(F)**.

To further investigate the variables associated with clinical trials in the field, trials with a “not applicable” phase were selected due to their distinct prevalence among trial groups. Subsequently, only the completed trials were chosen for further analysis (*n* = 92). The planned sample size, reflecting the anticipated number of participants at the trial's initiation, was gathered (Figure 2A). Additionally, the final sample size of patients actually included was also collected (Figure 2B). Finally, the difference between the anticipated and final sample sizes was calculated and displayed as a percentage of the anticipated sample size (Figure 2C). A substantial fraction of the completed trials recruited a lower number of participants than expected, which, in some cases, differed by up to 90%. Nonetheless, a moderate number of trials (*n* = 12) met or surpassed their initial enrollment targets, with some enrolling up to 50% more participants than anticipated.

The same approach was applied to analyze the duration and delay of the studies. The planned study duration, representing the expected duration estimated at the beginning of the clinical trial, was collected (Figure 2D). Subsequently, the final study duration, indicating the actual duration of the trial reported upon study completion, was also collected (Figure 2E). The difference between the final study duration and the planned study duration was calculated and reported as a delay. The observed delay for each trial was further used to estimate the average trial delay for completed trials in the field (10.6 months, Figure 2F). Given an average anticipated trial duration of approximately 40 months, the observed delay represents a 25% extension of the planned trial duration.

### 3.3 The trials delay is already measurable when reaching primary outcome

**Figure 3:**
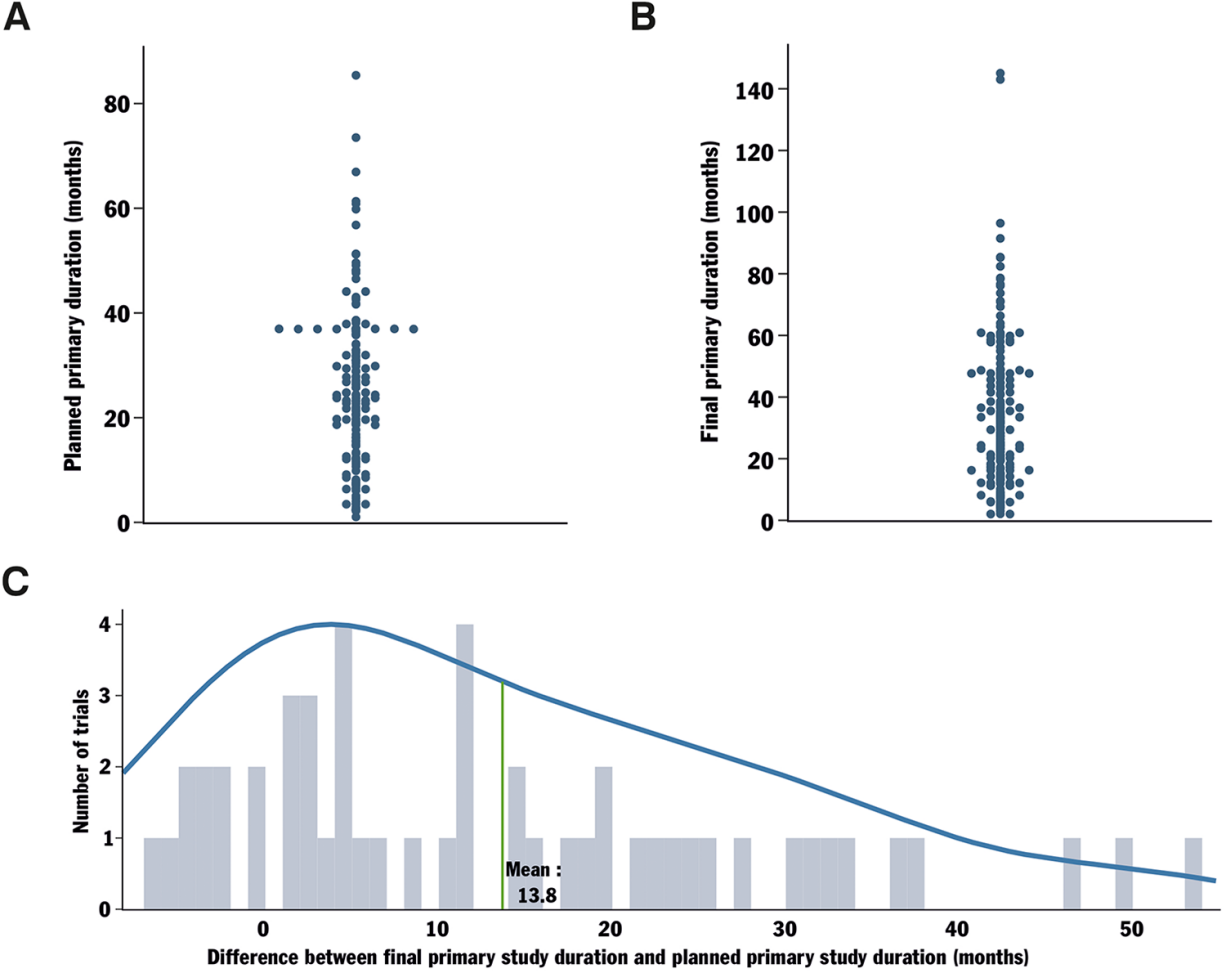
Analysis of the delay in reaching the primary completion date for trials with not applicable phase and their locations. Protocol AI further analyzed the data collected for trials with not applicable phase by investigating their planned primary duration (start date to planned primary completion date, **A)**. The final primary duration (time from start date to the actually registered primary completion date) was also analyzed **(B)**. The delay in reaching the planned primary completion date was calculated by comparing the originally planned primary duration of the trial to the final primary duration **(C)**.

To complete the duration and delay analysis on completed, not applicable phase trials, the planned primary duration of the trials was gathered (Figure 3A). The planned primary duration is the expected duration required to obtain the last data point for the primary outcome, collected from the last enrolled participant, as estimated at the beginning of the clinical trial. The same data were collected regarding the final primary duration, representing the actual time taken by each trial to obtain the last data point from the last enrolled participant (Figure 3B). From there, the primary study duration delay was calculated by determining the difference between the final primary study duration and the planned primary study duration (Figure 3C). The observed delay for trials to reach the primary outcome averaged 13.8 months, exceeding the overall clinical trial delay. Thus, delays within these trial groups are evident early in the trial process. This observation is consistent with the fact that many terminated trials in the field reported severe recruitment problems and could indicate a connection between recruitment difficulties and challenges in reaching the primary outcome.

Upon analyzing the trial locations, we noted that the majority of trials with a not applicable phase were hosted in the USA and China, with Germany following closely (Figure 3D). Simultaneously, phase 2, 3, and 4 trials were predominantly conducted in the USA (Supplementary Figure S2). These results may suggest a growing interest in China in the development and/or investigation of devices for spinal fusion surgery.

### 3.4 The impact of primary outcome on trial variables

**Figure 4:**
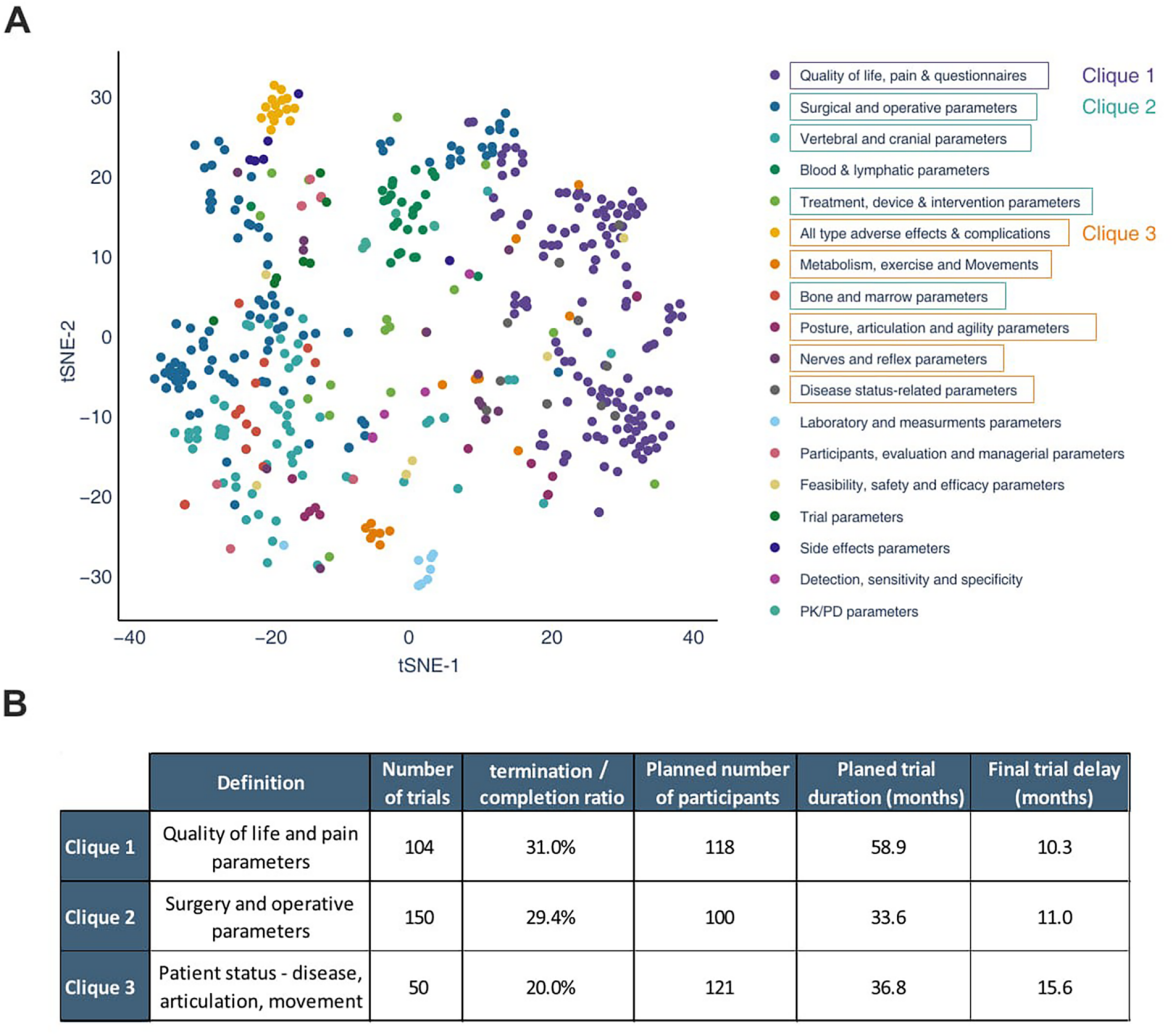
Analysis of the potential impact of the primary outcome on the probability of a trial reaching completion, on its sample size and on its observed delay. Using the data previously retrieved from trials with the not applicable phase, Protocol AI clustered their registered primary outcomes based on similarity. The 18 most important outcomes and their clusters of similarity are presented using a t-sne projection; there, a few clusters were grouped into cliques, allowing for mathematical comparison of the different outcomes **(A)**. The results of the comparison are presented in the table **(B)**.

Following the results indicating important delays to reach primary outcome, we performed a primary outcome cluster analysis using Protocol AI on trials with not applicable phase. We then compared the variables and performances associated with different primary outcomes. The registered primary outcome of each trial was automatically clustered by similarity analysis confirmed by two independent experts, and the 18 most prominent primary outcome clusters were depicted in a T-SNE projection (Figure 4A, Supplementary Figure S3). Interestingly, the most common measurement performed in the field was related to quality of life and pain, prior to surgical parameters. In fact, quality of life was the most commonly measured outcome among all (Supplementary Figure S3). It is worth noting that quality of life was also highly present as a secondary outcome among trials measuring another parameter as primary outcome (data not shown). Within the most commonly observed clusters of primary outcomes, three different concepts were created, grouping similar clusters into three cliques. The first clique consists of primary outcomes measuring patient-reported outcome measures. The second clique consists of treatment-related measurements, including surgical parameters and all vertebral, cranial and bone parameters. Finally, the third clique consists of measurements related to the patients' physiological and functional health assessments, encompassing complications, movement and posture parameters, in addition to reflexes, agility, and disease-related parameters.

The variables associated with the different cliques were analyzed (Figure 4B). It was observed that clique 3 presented a lower termination/completion ratio (20%) compared to the two other cliques (31% and 29%). However, trials in clique 3 planned the highest average number of participants (*n* = 121), while the completed trials in the clique faced the most significant delays (15.6 months). Moreover, the average planned trial duration of clique 3 is relatively short, particularly when compared to clique 1. The variability between the cliques may be partly explained by the impact of the primary outcome on the performance of the trials. Trials measuring pain, quality of life, or surgical parameters (clique 1 and 2) would be associated with a higher likelihood of termination compared to trials focusing on physiological and functional health assessments (clique 3). Despite having the highest completion rate, trials in clique 3 face the most significant delays. This highlights the impact of different primary outcomes on trial success. Given the limited number of completed trials in this phase of the analysis, the results exhibit high variability and should be interpreted with caution.

## 4 Discussion

The delays faced by clinical trials play a critical role in both the costs and the probability of a study's success (6, 18). However, this subject remains poorly documented, and the key reasons behind significant delays and trial failures remain unclear.

In this study, we investigated the delays encountered by trials on spinal fusion and their associated variables using Protocol AI. Following validation by independent experts, all data presented in the figures were automatically generated within seconds and remained fully accessible for further filtering and refinement. The software's flexible features significantly expanded experts' ability to investigate the historical impact of parameter variability on trials while allowing for in-depth feasibility assessments in record time.

Spinal fusion has garnered a steadily growing interest within the scientific community in recent years, notably due to the development of novel devices. This trend is corroborated by Protocol AI analysis, highlighting a sharp increase in the number of trials conducted annually on that topic, and by the fact that most of these trials do not possess an applicable phase (N/A phase). This notable trend in recent years, along with the rising number of trials categorized under this particular phase, called for a more in-depth analysis. Although the N/A classification is likely due to the medical device nature of these trials, they still represent varying levels of clinical trial stages, ranging from early safety testing (phase 2) to post-market studies. This broad variability consequently impacts results interpretability, limiting the generalizability of our observations.

Nevertheless, the analysis of the different phases revealed a remarkably consistent termination/completion ratio, hovering around 25%. Notably, 43% of all terminated trials cited participant enrollment as the primary reason for discontinuation. Even among completed trials with no applicable phase, over half failed to reach their expected participant numbers. These significant challenges in recruitment appear to play a pivotal role in the difficulties faced by trials focusing on spinal fusion. Surprisingly, the observed delay in completing the study, while substantial, was shorter than the delay reported in reaching the primary outcome (last patient, last data registration: 13.8 months vs. 10.6 months).

In addition, we observed that trials with not applicable phase not directly investigating spinal and operative parameters predominantly centered their primary outcome on quality of life and pain. Interestingly, this group of trials performed comparably to the field's average. However, a smaller clique of trials with a primary focus on patient status and movement parameters as the primary outcome faced more pronounced delays in completed trials compared to the two other major trial groups. This clique had the highest planned number of participants but also demonstrated the highest completion ratio among the three cliques. While this may initially seem counterintuitive, these results suggest that trials focusing on patients' status could endure longer delays. However, this extended duration could ultimately increase the likelihood of trial completion rather than termination. These results could indicate that, overall, trials measuring patients' status designed their protocols with greater perseverance than others: they demonstrated flexibility in adapting to issues, bending without breaking, and resisting termination. However, this conclusion should be considered with caution, being based on a limited number of completed clinical trials and a high variability in trial delays.

While the analysis offers unique insights into the delays witnessed in spinal fusion trials, determining the impact of the choice of primary outcome on trial performance remains challenging. Nevertheless, our findings underscore a strong association between delays observed in trials on spinal fusion and the challenges associated with patient recruitment. This finding may not be exclusive to spinal fusion trials.

Ambrosio et al. have notably highlighted the important impact of the trial funding source (private vs. public) on the outcome and performance of trials conducted on spinal fusion (19). Investigating the impact of additional trial variables—such as eligibility criteria, cohort size, recruitment rate, or reimbursement policies—on trial delays would require a substantially larger dataset to enable a robust statistical analysis, given the high variability of delays observed in clinical trials. Nonetheless, such a study would provide valuable insights for designing future clinical trials by identifying key parameters with the greatest influence on trial outcomes. Protocol AI, along with other novel cutting-edge tools for rapid and automated analysis of large-scale clinical trial datasets, could offer a transformative approach to examining the correlation and impact of multiple parameters on clinical trial success.

Research has demonstrated that investigators often overestimate the available patient pool for clinical trials (20). This analysis underscores the importance of delays in the field and the need to substantially reduce the recruitment burden to enhance cost-effectiveness and the likelihood of trial success. The data suggest that the implementation of a robust clinical trial recruitment plan and enforcement of stringent recruitment policies could markedly enhance the success rate of the trials in spinal fusion. Given the frequency of delays, it would be prudent to constitutively include the possibility of consequent delays in the trial timeline and include this estimate in the trial budget. It may be helpful and reasonable for completing the trial to consider planning of a potential trial delay for at least 25% of its estimated duration, rather than relying solely on an optimistic timeline. To ascertain the feasibility of adhering to the timeline, it appears essential to regularly evaluate the progress towards enrollment goals and the projected completion date. Additionally, the trial protocols could systematically include specific time points and enrollment goals. If these benchmarks are not achieved, the trial team should conduct a thorough analysis of the protocol and promptly engage in the execution of potential optimization processes. Should the goals remain unmet, responsible parties should consider terminating the clinical trial to mitigate the risk of unnecessary efforts dedicated to a failing trial. These actions could support researchers in assessing trial performance while potentially helping responsible parties accurately determine if and when a trial should be terminated.

The causes of trial delays are numerous and complex, certainly not reducible to individual factors. The data presented in this article focus on a limited number of trials for a very specific field, thus only highlighting trends in variables potentially causing delays and failures. Nevertheless, delays in trials lead to increased resource usage and costs, directly affecting the trial's probability of success. Furthermore, delays postpone the delivery of novel therapies to patients, posing ethical concerns. In the era of modern technologies, dedicated efforts should be channeled towards swift and efficient patient recruitment, while avoiding the pitfall of overestimating the available patient pool. This seemingly simple yet pivotal initiative could fundamentally reshape the likelihood of success for spinal surgery trials, benefiting all stakeholders involved.

## Data Availability

The raw data supporting the conclusions of this article will be made available by the authors, without undue reservation.

## Supplementary material

Supplementary Figure S1 Overview of inclusion and exclusion criteria of trials. The criteria consist of both Protocol AI automated procedures and experts' revision processes.

Supplementary Figure S2 Location of clinical trials based on phases. The clinical trials registered as phase 2, 3, 4 and ‘Not Applicable’ presented in Figure 1 were further analyzed by Protocol AI. The location of each trial was presented using a world map.

Supplementary Figure S3 Primary outcome analysis and quantification for spinal fusion trials with the not applicable phase. Protocol AI sorted and clustered all the primary outcomes of the spinal fusion trials with not applicable phase. The clusters are presented as columns being ranked according to their number of appearances.
