# Supplementary figures and images for: Work smart, not hard: analysis of delays faced by clinical trials investigating spinal fusion using Protocol AI
Source: Front Surg. 2025 Mar 27;12:1546367. doi: 10.3389/fsurg.2025.1546367 (PMC11983609; doi:10.3389/fsurg.2025.1546367)

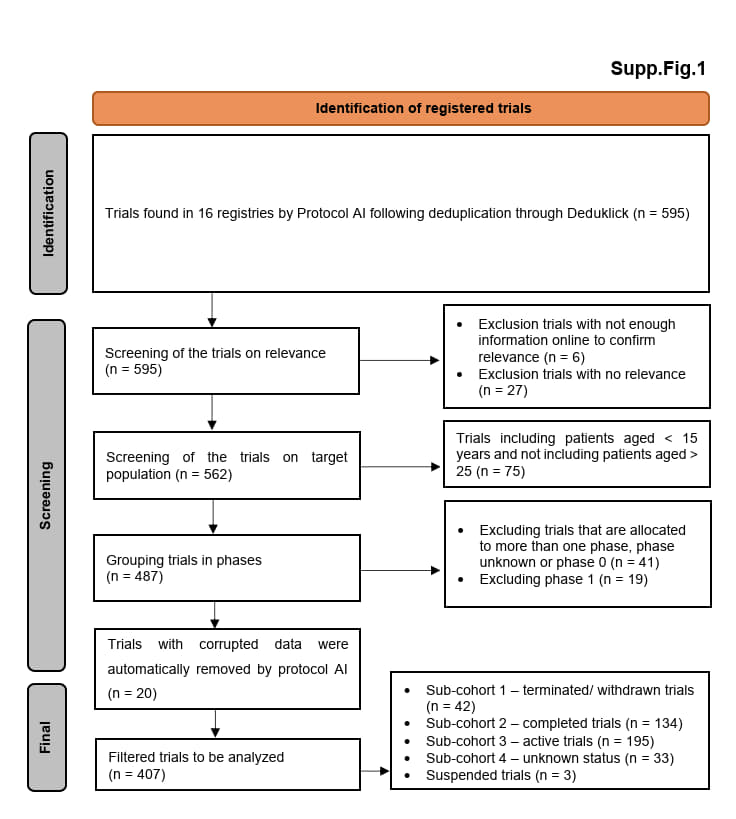

Supplement: Supplementary Figure S1 — Overview of inclusion and exclusion criteria of trials. The criteria consist of both Protocol AI automated procedures and experts' revision processes. [file Image1.jpeg]

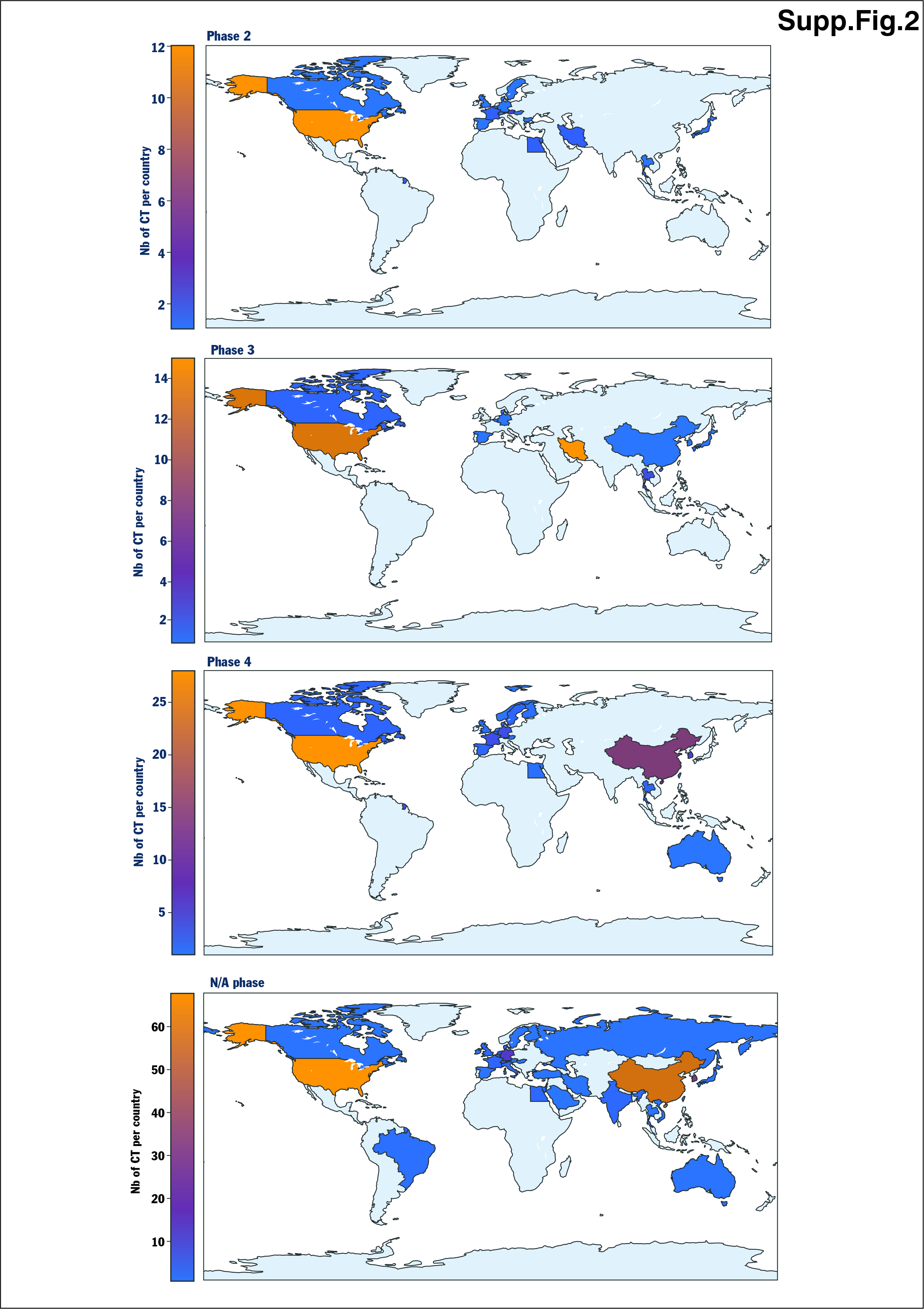

Supplement: Supplementary Figure S2 — Location of clinical trials based on phases. The clinical trials registered as phase 2, 3, 4 and ‘Not Applicable’ presented in Figure 1 were further analyzed by Protocol AI. The location of each trial was presented using a world map. [file Image2.jpeg]

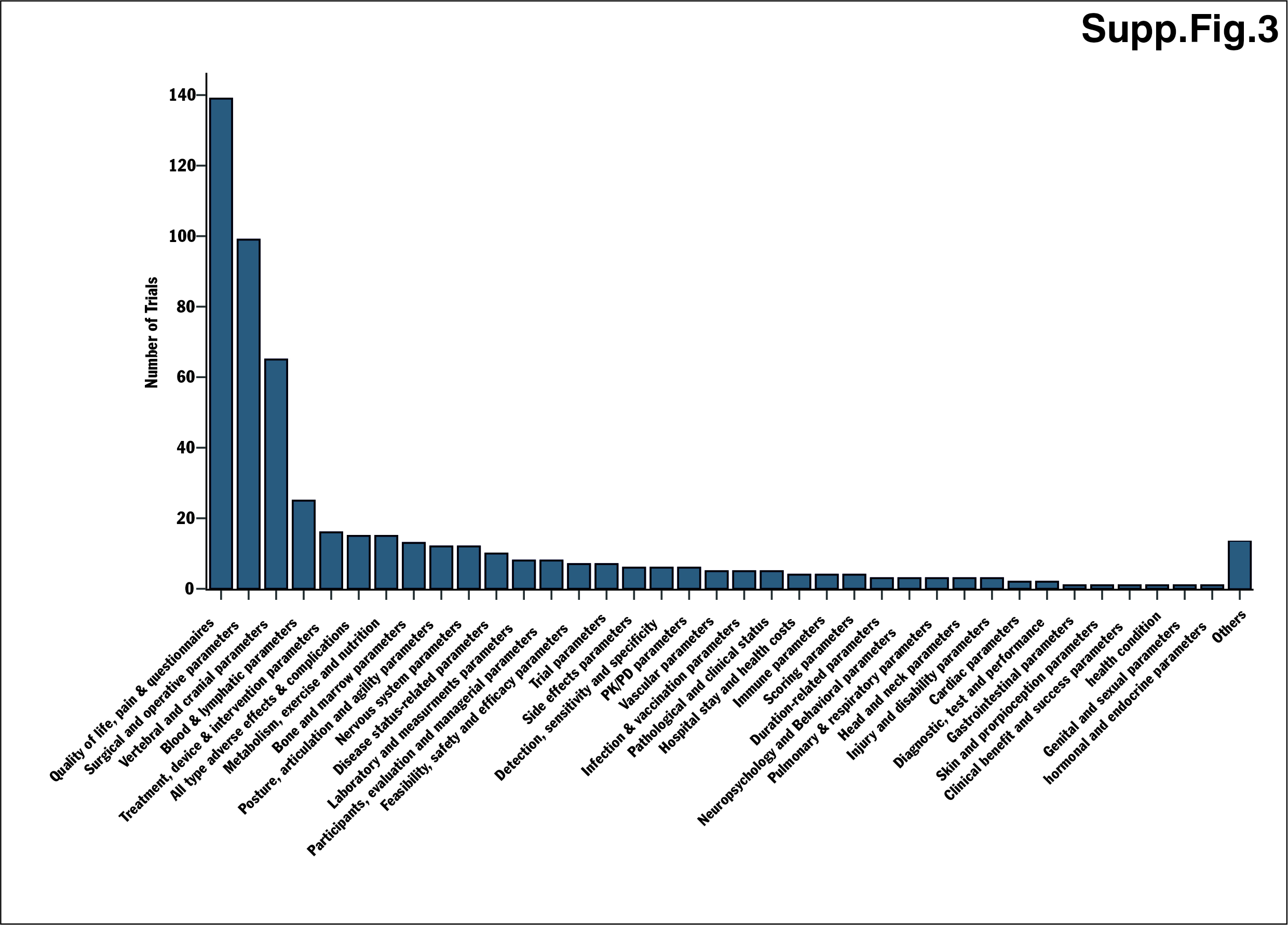

Supplement: Supplementary Figure S3 — Primary outcome analysis and quantification for spinal fusion trials with the not applicable phase. Protocol AI sorted and clustered all the primary outcomes of the spinal fusion trials with not applicable phase. The clusters are presented as columns being ranked according to their number of appearances. [file Image3.jpeg]
